# Supplementary figures and images for: Imported malaria in Cabo Verde (2010–2024): Risks to post-elimination stability
Source: PLOS Glob Public Health. 2025 Jun 12;5(6):e0004592. doi: 10.1371/journal.pgph.0004592 (PMC12161540; doi:10.1371/journal.pgph.0004592)

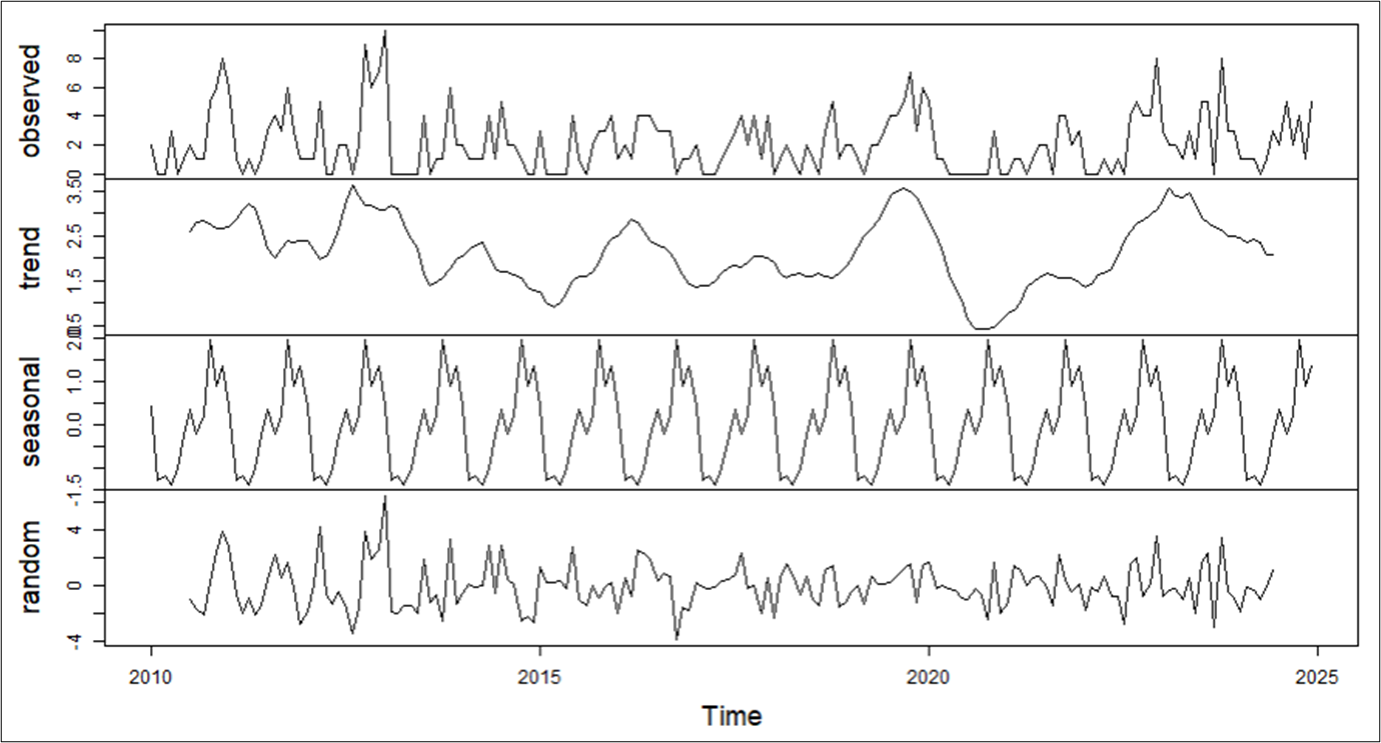

Supplement: S1 Fig — (TIFF) [file pgph.0004592.s002.tiff]

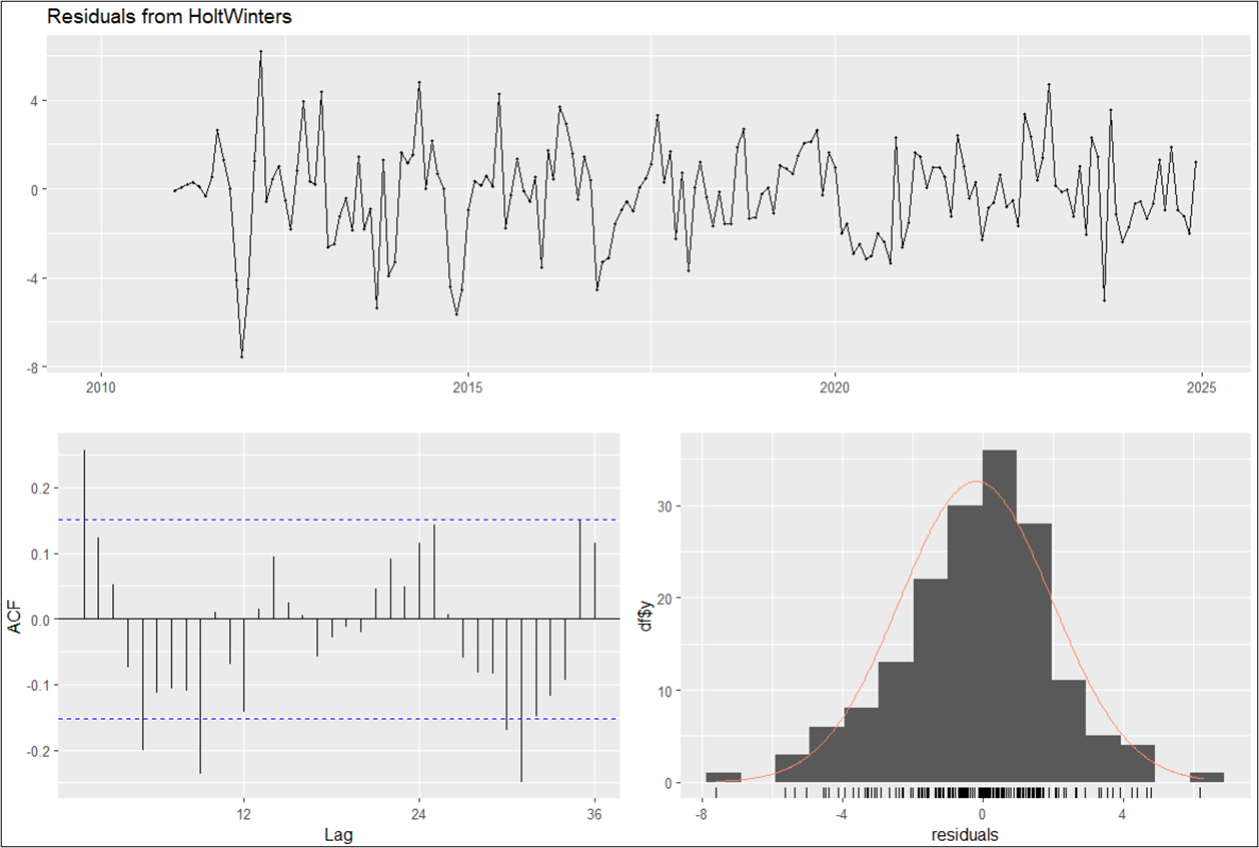

Supplement: S2 Fig — (TIFF) [file pgph.0004592.s003.tiff]

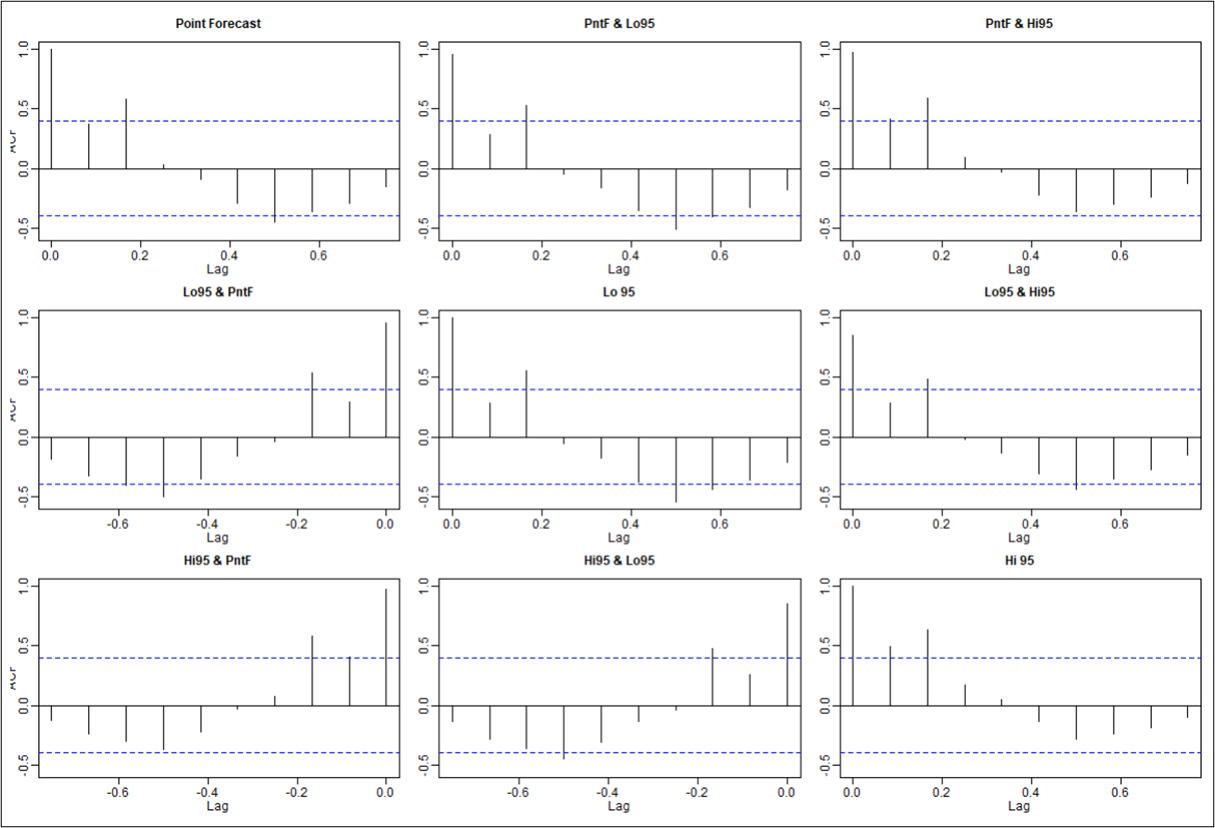

Supplement: S3 Fig — (TIFF) [file pgph.0004592.s004.tiff]
