# Supplementary material for: Imported malaria in Cabo Verde (2010–2024): Risks to post-elimination stability
Source: PLOS Glob Public Health. 2025 Jun 12;5(6):e0004592. doi: 10.1371/journal.pgph.0004592 (PMC12161540; doi:10.1371/journal.pgph.0004592)
Supplement: Attachment — Submitted filename: Point-by-Point REPONSE TO THE REVIEWERS.docx [file pgph.0004592.s007.docx]

**REVIEW I**

**PGPH-D-25-00516
Characterization of Imported malaria in Cabo Verde, 2010-2024: A challenge in preventing the disease re-establishment in the country
PLOS Global Public Health**

Dear Dr. DePina,

Thank you for submitting your manuscript to PLOS Global Public Health. After careful consideration, we feel that it has merit but does not fully meet PLOS Global Public Health’s publication criteria as it currently stands. Therefore, we invite you to submit a revised version of the manuscript that addresses the points raised during the review process.

The first area of focus for the revision should be the presentation clarity. This includes the definition of terminology which should be consistent with what is presented in the tables. Secondly, as mentioned by a reviewer, the methods (joinpoint regression and time series analysis) should be described in such a manner that it is reproducible. To that end, it would be good to detail the data processing, methods (including model selection and goodness-of-fit measures) and results.   

Please submit your revised manuscript by May 22 2025 11:59PM. If you will need more time than this to complete your revisions, please reply to this message or contact the journal office at [globalpubhealth@plos.org](mailto:globalpubhealth@plos.org). When you're ready to submit your revision, log on to <https://www.editorialmanager.com/pgph/> and select the 'Submissions Needing Revision' folder to locate your manuscript file.

Please include the following items when submitting your revised manuscript:

- A rebuttal letter that responds to each point raised by the editor and reviewer(s). You should upload this letter as a separate file labeled 'Response to Reviewers'.
- A marked-up copy of your manuscript that highlights changes made to the original version. You should upload this as a separate file labeled 'Revised Manuscript with Track Changes'.
- An unmarked version of your revised paper without tracked changes. You should upload this as a separate file labeled 'Manuscript'.

Guidelines for resubmitting your figure files are available below the reviewer comments at the end of this letter.

We look forward to receiving your revised manuscript.

Kind regards,

**Point by Point Response**

The authors would like to thank the journal, the editor, and the reviewers for their work. Several aspects have been mentioned here that reinforce the quality of the manuscript and its scientific nature, among other things.

The authors thoroughly reviewed the questions one by one, making any necessary adaptations, to respond to all the questions and suggestions raised.

Most of the questions/observations were absorbed. Additional information was adjusted, and in some cases, we reinforced the initial idea, following the objective of the proposed article.

Regarding the methodology, the authors reinforced the initial idea in some respects by responding to the reviewers' questions and proposals.

We thank you for reading the AUTHORS' RESPONSE (**AR**) to the questions/suggestions below.

**Journal Requirements:**

1. We note that your Data Availability Statement is currently as follows: All data are available on the manuscript.
 
 Please confirm at this time whether or not your submission contains all raw data required to replicate the results of your study. Authors must share the “minimal data set” for their submission. PLOS defines the minimal data set to consist of the data required to replicate all study findings reported in the article, as well as related metadata and methods (<https://journals.plos.org/plosone/s/data-availability#loc-minimal-data-set-definition>).
 
 For example, authors should submit the following data:
 
 - The values behind the means, standard deviations and other measures reported;
 - The values used to build graphs;
 - The points extracted from images for analysis.
 
 Authors do not need to submit their entire data set if only a portion of the data was used in the reported study.
 
 If your submission does not contain these data, please either upload them as Supporting Information files or deposit them to a stable, public repository and provide us with the relevant URLs, DOIs, or accession numbers. For a list of recommended repositories, please see <https://journals.plos.org/plosone/s/recommended-repositories>.
 
 If there are ethical or legal restrictions on sharing a de-identified data set, please explain them in detail (e.g., data contain potentially sensitive information, data are owned by a third-party organization, etc.) and who has imposed them (e.g., an ethics committee). Please also provide contact information for a data access committee, ethics committee, or other institutional body to which data requests may be sent. If data are owned by a third party, please indicate how others may request data access.

AUTHORS' RESPONSE (**AR**) : The authors confirm that all the data used was inserted in the first draft and that no other data was used.

2. Figures 4 and 6: please (a) provide a direct link to the base layer of the map (i.e., the country or region border shape) and ensure this is also included in the figure legend; and (b) provide a link to the terms of use / license information for the base layer image or shapefile. We cannot publish proprietary or copyrighted maps (e.g. Google Maps, Mapquest) and the terms of use for your map base layer must be compatible with our CC-BY 4.0 license.

Note: if you created the map in a software program like R or ArcGIS, please locate and indicate the source of the basemap shapefile onto which data has been plotted.

If your map was obtained from a copyrighted source please amend the figure so that the base map used is from an openly available source. Alternatively, please provide explicit written permission from the copyright holder granting you the right to publish the material under our CC-BY 4.0 license.

Please note that the following CC BY licenses are compatible with PLOS license: CC BY 4.0, CC BY 2.0 and CC BY 3.0, meanwhile such licenses as CC BY-ND 3.0 and others are not compatible due to additional restrictions.

If you are unsure whether you can use a map or not, please do reach out and we will be able to help you. The following websites are good examples of where you can source open access or public domain maps: 
* U.S. Geological Survey (USGS) - All maps are in the public domain. (<http://www.usgs.gov>) 
* PlaniGlobe - All maps are published under a Creative Commons license so please cite “PlaniGlobe, <http://www.planiglobe.com>, CC BY 2.0” in the image credit after the caption. (<http://www.planiglobe.com/?lang=enl>) 
* Natural Earth - All maps are public domain. (<http://www.naturalearthdata.com/about/terms-of-use/>)

**AR**: All sources from the shape files are already present at the legend of figures 4 and 6? Maybe add the following “World countries shapefiles were downloaded from the ArcGIS Hub (url: <https://hub.arcgis.com/datasets/esri::world-countries-generalized/explore>; Terms of use: <https://goto.arcgis.com/termsofuse/viewtermsofuse)>.”

**Reviewers' comments:**

**Reviewer #1: Summary:**
This paper examines malaria cases in Cabo Verde over a 14-year period leading up to 2024, when the country achieved WHO certification for malaria elimination. During this period, Cabo Verde operated as a near-elimination setting, primarily focusing on the challenge of imported cases. The analysis includes calculations of incidence, mortality, and case-fatality rates, along with trend analysis using joinpoint regression. Publishing these findings is valuable for other countries approaching elimination, as it may provide insights on achieving and maintaining this goal.

Despite a few minor discrepancies in the results section, I recommend this paper for publication.

**AR**: Thank you for your recommendation

MAJOR COMMENTS:

Results
Table 1 and First Paragraph:
In the text, you mention 441 local cases in 2017, but in Table 1, this is broken down into 423 local cases and 18 relapsed cases. It’s important to clarify the terminology used if relapsed are also local or not and update both the text and the table accordingly.

**AR**: Corrected. All the relapsed cases are the local cases.

Second Paragraph:
The authors state that after 2017, the country began registering only imported cases, but this is not reflected in Table 1. There is one local case in 2018 and 11 in 2014, which is also mentioned in the following paragraph. Please revise or remove this statement. Additionally, the authors mention that after 2018, it became possible to more accurately record imported cases, with only one case per year. However, in Table 1 there are more than one per year. Please check the text and Table 1 to ensure they align and clearly define "introduced" and "relapsed" cases.

**AR**: Corrected. The information in this paragraph is related to the introduced cases.

MINOR COMMENTS

Abstract
- Please spell out all acronyms and, where possible, avoid using them in the abstract.
- Angola (N = 72; 18.6%%) – additional percentage should be removed.

**AR**: Corrected.

Introduction
Paragraph 4
- The second sentence is unclear. Please revise: “There were times when the country interrupted local cases transmission.”
- In the same sentence you mention there were times when the country interrupted local transmission could you provide any evidence in the form of citations for these.
- In the third sentence, please clarify or correct the phrase “Immediately after the outages.” What do you mean by "outages"?

**AR**: Corrected. Sentence clarified. Citation added and outbreak corrected as interruption of malaria cases.

Methods
Study design - Paragraph 2
- The total population is written as 511.534; I assume this should be 511,534.

**AR**: Corrected.
- When describing where most people live, you mention these areas have better climatic and relief conditions. Please clarify why they are considered better—are they more temperate and less mountainous?

**AR**: Corrected.
- When describing the country's demography, could you include references for age, population growth rate, and life expectancy?

**AR**: Reference added.
- Please clarify which portion of 2024 the data covers. Is it for the complete year but unverified, or are there missing months?

**AR**: The paragraph was clarified. The data covers the full year of 24, with validated data.
Data processing and statistical analyses
- Why have you have used the term lethality rate as opposed to case fatality rate?

**AR**: Corrected for case fatality rate.

Results
Table 2:
- In the title, please clarify if this refers to Imported Malaria Cases in Cabo Verde.
- I recommend restructuring this table to focus on the characteristics of imported cases, and separating the data for incidence, mortality, and lethality rates.
- Correct the spelling of "sex" (not "sexe").

**AR**: Corrected.

Figure 2:
Is this a form of stacked chart? Please specify in the title or figure caption if it is.
**AR**: Corrected. Specified in the title.

Figure 4:
The percentages and their corresponding colours difficult to follow. Consider using a continuous colour scale, and perhaps include a supplementary table with the exact percentages.

**AR**: Corrected.

Figure 5:
Countries are listed on the x-axis, but for Guinea, the capital, Conakry, is also included – please standardise this.

**AR**: The idea is to differentiate the different Guinea countries, namely: Guinea-Bissau, Guinea-Conakry, Equatorial Guinea.

Discussion
Third Paragraph:
- Please define all acronyms, such as ECOWAS.
- In the sentence, “In addition to the ‘brother countries,’ neighbouring countries such as Senegal, a member of ECOWAS, and Guinea-Bissau and Cape Verde” – it suggests that Cape Verde is a neighbouring country to itself, which is confusing. Please clarify.
- The last sentence of the third paragraph is difficult to understand. Please improve clarity.

**AR**: Corrected and information updated.

Ninth Paragraph:
- Please add references when discussing the interval between symptoms and visiting healthcare.

**AR**: Reference added.

Tenth Paragraph:
- Please define all acronyms, such as KAP.

**AR**: definition added.

Second to Last Paragraph:
- Use a lowercase "p" for Prediction.
- "So." should not be a sentence on its own.
- "Now, rather than ever" should be changed to "Now more than ever."

**AR**: Suggestion accepted and updated.

**Reviewer #2:** First, thank you to the authors for this valuable and timely manuscript. The paper is well-organized, rich in surveillance data, and offers important insights into post-elimination malaria risks in Cabo Verde. This topic is highly relevant for other recently malaria-free countries grappling with the challenge of imported cases.

That said, a major revision is recommended to sharpen the focus, improve clarity, and enhance policy utility. Please see detailed comments below.

Major Comments –
1. Overly descriptive, needs more analytical depth
The manuscript tends to summarise data without offering strong interpretation or synthesis.

For example, trends in figures and tables are restated in the text without drawing broader conclusions or identifying actionable implications.

Suggested Fix: Strengthen the discussion of what the observed trends mean for malaria reintroduction risk and preparedness strategy.
**AR**: We appreciate the comment, but the authors respectfully disagree that the ‘overly descriptive’ is a negative point. We feel this undermines much of the effort we dedicated to this work. Yet, we have changed the discussion to address reintroduction risk and preparedness strategies. We appreciate your suggestion.

2. Too lengthy and repetitive in sveral sections
The introduction and discussion include repetitive paragraphs, particularly around WHO certification, climate change, and the need for sustained vigilance.

Suggested Fix: Reduce length by eliminating repetitive phrases and focusing on unique contributions of the study.
**AR**: We appreciate the comment, and we have revised the text in a way the makes it more objective and concise.

3. Missing or underdeveloped aspects
Delay in care-seeking behavior (average of 4.2 days for imported cases) is mentioned, but not analysed in-depth. Why is this delay occurring, and what are the implications?
**AR**: The idea was updated in the discussion section.

The gender and age profile (82% male, >90% over 25) is noted but not discussed—these trends could inform targeted interventions for high-risk groups.
**AR**: Thank you for the input. We have inserted discussion topics related to it.

There is little discussion on P. ovale or mixed infections, though small in number, these may impact treatment approaches.

**AR**: We agree, we have added a discussion on this topic in lines 442-447”

4. Link between imported cases and local reintroduction needs more emphasis
The paper acknowledges that 16 introduced cases were reported in 2024 but does not analyse how they may be linked to specific importation events.

Suggested Fix: Consider adding a case example or flow pathway illustrating how an imported case might lead to onward transmission.

**AR**: We appreciate your comment. We have added an example of this situation while discussing with available literature.

5. Figures and tables need condensation
Several figures (especially those showing month-wise or origin-wise data) could be combined or moved to supplementary materials.

Simplify visuals and keep the focus on Figures 1 (trend), 3 (forecast), and 4 (geographic clustering).
**AR**: We appreciate your comments,

a) But we believe that the input of those tables and figures as it is strengthen transparency and facilitates readability, as we understand that not every reader dedicates time to check for supplementary files.

Minor Comments
Title: Consider simplifying to: “Imported Malaria in Cabo Verde (2010–2024): Risks to Post-Elimination Stability”.

**AR**: Suggestion accepted and updated.

Abstract: Rephrase lines 34–35 – forecast ranges (-23.1 to 84) suggest low model certainty. Consider framing more cautiously.

**AR**: Suggestion accepted and updated.

Line 146–158 (Study area): This could be shortened or partially moved to supplementary material.

**AR**: The authors think that the “Study rea “is already a short paragraph with only 13 lines, with the description about the country, which does not have information for the supplementary material.

Line 300+: The discussion about climate change is helpful but currently too generic. Trim it and cite relevant local studies or specific Cabo Verde projections if available.

**AR**: There is no specific study regarding climate change and the VBD in Cabo Verde. The authors opted for generic information because it may apply to island countries like Cabo Verde.

Check small inconsistencies: e.g., Angola mentioned as both contributing 18.6% and 19.0% (Lines 37 and 550).

**AR**: Data corrected and updated.

**Reviewer #3: SUMMARY**

The authors have conducted a study on characterising imported cases of malaria in Cabo Verde and presented a comprehensive discussion on the challenges and priorities for preventing the disease re-establishment in the country. The methods are clearly described and appropriate to the analysis. The results are well presented, with the data and analyses supporting all results. The results are contextualised in relevant literature with a good discussion on the implications of the findings. I commend the authors on an interesting and important piece of work.

While the manuscript is generally well written and easy to follow, it would benefit from a proofread and revision of a few sentences that are difficult to interpret. I have highlighted some of these examples below.

PROOF-READING

Line 63 - Should “Indigenous” be capitalised?
Line 77 - “Outages” seems like an odd choice of words
Line 117 - I suggest removing “the” so the sentence reads more easily: “This will support decision-makers…”
Line 129 - This should be a comma to denote a thousand rather than a period (I see you have used commas lower down so presumably this is a typo). Also please check for consistency of using either a comma or space to denote thousands and a period to denote decimal points (e.g. in Table 2 you are using a comma to denote decimal points.)
Line 177 - I suggest using the term “Case fatality rate” instead of “Lethality rate” (also in Table 2) as it is a more commonly used term. Also revise this in Table 2.
Line 207 - Full stop missing at the end of the sentence
Table 2 - English spelling: Cases by sex (not sexe) and Mix or Mixed (not Mixte)
Line 3 on page 14 - check whether you’ve introduced the acronym KAP yet.
Paragraph 3 on page 15 - No need to put ‘climate change’ in inverted commas.
General: Please do a read through of the document to check for unnecessary use of capitalisation in the middle of sentences.
General: Please check for consistency of using either lowercase or uppercase when referring to figures in the text.

**AR**: Thank you for the suggestions. All suggestions were reviewed and updated.


RESULTS

Suggest adding a footnote to Table 1 with the definitions of the 4 key terms (imported, local, introduced, relapsed).

**AR**: Table 1 added with the definition.

It seems that Table 2 is only providing details on Imported cases, not all cases. This should be made clear in the heading.

**AR**: Corrected.

When commenting on the species of imported cases: Which species are detected on the tests and do all tests used have the ability to detect different species?

**AR:** Yes. The country uses RDT Pf/Pan to distinguish *P. falciparum* from the other species. All cases are confirmed by microscopy, and all confirmed cases by microscopy are (re)confirmed by PCR.

“Over the years, the number…” - perhaps rephrase to “Over the remaining years…” for clarity.

**AR**: Corrected.

On page 10 when discussing Figure 1, do you have any thoughts on why 2018 was such a high year? It appears to be an anomaly and I was wondering whether there are potential explanations. This is just something I was curious about when reading, but does not need to be addressed if there is no clear comment to add to it.

**AR**: The high year is 2019 with 39 imported cases, not 2018 (with 18 cases). The high number of cases may be related to the high number of travels. The country started to receive more flights from endemic countries, and with the increase in tourism, it resulted in high malaria cases. It may have the same sense in the years after COVID-19, as we can see in 2023 and 2024, the tendency to have more imported cases. However, it is also related to the epidemiological surveillance that is sensitive to detecting real cases.

When presenting Figure 3, the predictions have a wide confidence interval. I think this should be raised in the limitations or addressed somewhere in the manuscript. Also, could the authors clarify what the interpretation of values below zero is for the CI when the unit is cases.

**AR**: We appreciate the reviewer’s careful attention to the interpretation of our forecast model and its associated uncertainty. We acknowledge that the 95% confidence interval (CI) depicted in Figure 3 is relatively wide, and we have now addressed this more explicitly in the limitations section of the manuscript. As is common in predictive modeling, particularly when projecting several years into the future, the CI reflects the compounded uncertainty over time. The width of the interval is a natural result of using a 95% confidence level, which is a standard threshold for balancing statistical confidence with interpretability.

With regard to the lower bounds of the CI occasionally falling below zero, we agree that this requires clarification. While the unit of analysis is count data (imported malaria cases), and negative case numbers are not possible in real-world settings, the model produces a normal approximation of the distribution of errors, which can yield negative values in the lower tail of the CI. These negative values should not be interpreted literally, but rather as an indication that the model predicts a high probability of observing zero cases in those time periods. We have now added this clarification to the figure caption and methods section.

In Figure 5, remove the decimal point in the Year legend.

**AR**: Corrected it.

In the results text above Figure 5: “Fewer countries contributed…” - It seems like there are 24 other countries identified as contributing to imported cases (i.e. quite a lot, not fewer). Please reword this for accuracy/clarity.

**AR**: Corrected.


DISCUSSION

Overall, the authors have presented a comprehensive discussion section raising important considerations, challenges, and priorities for maintaining malaria-free status. I think it would strengthen the discussion section to include a brief summary of the key finding at the beginning of the discussion; and to include a comment on limitations of the study.

**AR**: Improved and comment on limitations added.

While the discussion section is generally well written and easy to follow, there are a few sentences that need revision. I have shown these below - please could the authors rewrite to improve clarity and check grammar.

Pg 12 paragraph 1: “In addition to the “brother countries”, neighbouring countries such as Senegal, a member of ECOWAS, and Guinea-Bissau and Cape Verde, which have an open border with the archipelago, is a destination of choice.”

**AR**: Corrected.

Pg 12 paragraph 1: “It is known that livelihood activities and population mobility influence the epidemiology of vector-borne disease and, which may increase the risk of malaria cases are barriers to and influence malaria risk in elimination settings [30,31].

**AR**: Corrected.

Pg 12 paragraph 2: Suggest minor changes to improve readability (using ‘most’ three times in the sentence makes it hard to digest). Change: “Similarly to elsewhere on the African continent, most of the imported malaria cases originated from some of the most African malaria-endemic countries, where P. falciparum is the most predominant parasitic species in humans [38,39].” To: “Similarly to elsewhere on the African continent, the majority of the imported malaria cases originated from some of the most malaria-endemic African countries, where P. falciparum is the predominant parasitic species in humans [38,39].

**AR**: Corrected.

Pg 12 paragraph 3: In: “1- the “importation of cases” of malaria, from boats that stop by the endemic malaria countries before achieving the archipelago and docking in its ports, especially in São Vicente.” I suggest changing ‘achieving’ to ‘reaching’ or ‘arriving at’.

**AR**: Corrected.

Pg 15 paragraph 3: “With rising temperatures and competent vectors present, there is a risk of their infestation by infected travellers besides the widespread insecticide resistance.”

**AR**: Corrected.

Pg 15 paragraph 3: “So. Now, rather than ever, Cabo Verde should reinforce active imported cases surveillance and not ‘relax’ due to the low absolute number of cases.”

**AR**: Corrected.

**Reviewer #4:** This manuscript presents a description of surveillance data and malaria importation over a 15 year period in Cabo Verde, during which malaria elimination was certified. The paper is a useful commentary on the challenges of maintaining local elimination and will be useful for other countries with similar goals.

Main comments
- Methods, line 142-144. What was the method used to determine the location an infection was acquired? I would expect that the definition would include a requirement for travel within a defined timeframe prior to the diagnosis.

**AR**: Corrected.

- Methods. I note that the terms introduced case and relapse case are used in the results, but definitions of these terms are not provided in the methods.

**AR**: A table with case definitions was included.
- Methods, data processing. It would be informative to have some further details on data collection and processing beyond the single sentence on line 175. Presumably case data are not recorded at health facilities in Excel? Alternatively, are there other publications on the surveillance system in Cabo Verde which could be cited here?

**AR**: Corrected and additional information added.

- I am not convinced that the joinpoint regression adds much to the manuscript. The methods and results are not presented in any detail, and this does not seem to add any further information over what could be interpreted in a descriptive analysis. I recommend either presenting these in more detail or removing.

**AR**: We respectfully disagree with the reviewer’s suggestion and would like to clarify the added value of the joinpoint regression in our analysis. While a descriptive assessment can offer an overview of trends in imported malaria cases, it does not formally identify statistically significant changes in trend direction or quantify the magnitude of change. Joinpoint regression, by contrast, provides an objective and statistically robust method to detect inflection points in temporal trends and to calculate the Annual Percentage Change (APC) for each segment.

This method is particularly important in our study context, where the post-pandemic period (2020–2024) shows a concerning reversal from previous declines. The joinpoint analysis allowed us to formally identify this increase and quantify it (+25.75% APC), something that would not be possible with descriptive statistics alone. Moreover, the method helps distinguish between routine year-to-year variability and statistically meaningful trend shifts—offering a more reliable foundation for public health interpretation and response.

In response to the reviewer’s comment, we have now expanded the Discussion section to interpret the key findings and their implications.

- Lines 190-103. Similarly, the time series forecasting is not presented in sufficient detail for the method to be replicable. Please either provide further details (e.g. how seasonality and “randomness” were accounted for in the model), the R packages used, any covariates, the unit of analysis (annual national data?) and any other assumptions or data handling procedures to develop and validate the model approach. Idem,

AR: We thank the reviewer for this important suggestion. In response, we have added a more detailed description of the forecasting methodology in the Methods section to improve transparency and replicability.

Briefly, we used the Holt-Winters exponential smoothing method for time series forecasting, implemented via the *forecast* package in R. Given the relatively low monthly counts of imported malaria cases, we opted for the **additive** Holt-Winters model, which is more appropriate than the multiplicative version when the magnitude of seasonal variation remains roughly constant over time. The model accounts for **seasonality**, **trend**, and **randomness (error)** components inherent to time series data. Specifically, the **randomness** or irregular component is modeled as the residual (unexplained) variance after accounting for level, trend, and seasonality, which is a standard feature of exponential smoothing models.

The prediction was conducted using **monthly national data**, as described in the Data Sources section, and projected forward for a 24-month horizon. No covariates were included in the model, as the primary goal was to generate short-term forecasts based solely on historical patterns of case notification.

These details have now been included to support the reproducibility of our analysis. We hope this addresses the reviewer’s concerns.

- Line 199. I believe that the “spatial model” mentioned in line 199 is more accurately reported as a descriptive analysis, unless there are any further spatial statistical methods used.

AR. Corrected.

Additional minor edits
- Abstract. Please ensure that the acronym APC is explained. The abstract should be interpretable as a stand-alone piece of information.

**AR**: Corrected.
- Abstract. “The most common countries of infection were…” could perhaps be better phrased as “the most common sources of imported infection were…” since a country is not itself infected.

**AR**: Corrected
- Introduction, lines 106-108. It may be helpful in this mention of different data collection and surveillance approaches to highlight that the country’s level of transmission is also relevant – the system in a moderate burden country is likely to be very different to that of a country close to elimination.

**AR**: Corrected and information updated.
- Introduction, line 112. “Over the years all cases registered in the country have been the subject of an epidemiological investigation”. It would be helpful to state the time period here. Is this a recent development or has it been going on for decades?.

**AR**: Corrected and information updated.
- Methods, line 176. Please confirm the source of your population denominator data (national census?) .

**AR**: Corrected
- Methods, line 177. “Case fatality rate” is a more commonly used terminology than “lethality rate”

**AR**: Corrected
- Line 206. If the data are publicly available as stated here, please identify the source so that other researchers may also have access to these data.

**AR**: Corrected and information updated.
- Line 223. Should this sentence refer to introduced cases rather than imported cases? **AR**: Corrected and information
- Results. Is a “semester” a transmission season, a 6-month period, or something else? **AR**: Yes.

**Reviewer #5:** The manuscript presents an interesting area of study and offers valuable lessons for malaria-eliminating countries. However, it's unclear how:
• the authors have defined the importation of cases mainly from outside the country, which is OK, but the analysis is retrospective. It would have been helpful to see an analysis of importation both between the islands within Cape Verde and from outside the country.

**AR**: We understand that importation between Island could be insightful, but the objective of this research was to address imported cases from other countries, as specified in the study population and in the imported cases definition in the Methods - Study design, population and period section. We will consider conducting an additional study on that matter. Thank you.

As it stands, the manuscript primarily suggests that imported cases are from outside the country, which is OK and aligns with WHO guidelines for preventing re-establishment following certification. However, since this is a retrospective analysis, it should address both in-country and external sources of importation. This will help identify the most vulnerable entry points or islands.

**AR**: We understand and appreciate the suggestions, although as indicated in the manuscript, the objective of this study is to examine malaria cases imported from outside Cabo Verde, in line with WHO recommendations for countries maintaining elimination status. An analysis of internal movement and in-country transmission dynamics would require a separate design and data sources, and is therefore beyond the scope of the current work.

• This is not described in the MS, Could the authors provide more detailed categories in their importation framework, such as the location of residence and the most likely location of infection?

**AR**: Unfortunately, this level of information is not available in the data sources used and provided by the Ministry of Health of Cape Verde.

• Additionally, the authors should check for spelling errors throughout the manuscript.

**AR**: We appreciate the comment. We have made an extensive review of English language in the manuscript. Thank you.
