# Supplementary material for: Imported malaria in Cabo Verde (2010–2024): Risks to post-elimination stability
Source: PLOS Glob Public Health. 2025 Jun 12;5(6):e0004592. doi: 10.1371/journal.pgph.0004592 (PMC12161540; doi:10.1371/journal.pgph.0004592)
Supplement: Attachment — Submitted filename: REVIEW_Response reviewers point by point.docx [file pgph.0004592.s008.docx]

Dear Dr. DePina,

Thank you for submitting your manuscript to PLOS Global Public Health. After careful consideration, we feel that it has merit but does not fully meet PLOS Global Public Health’s publication criteria as it currently stands. Therefore, we invite you to submit a revised version of the manuscript that addresses the points raised during the review process.

 As the reviewers point out, the difference between a malaria case and a confirmed malaria case is not clear and there is a need for further proof-reading. In addition, to facilitate reproducibility, it would be good to include details of the model selection/comparison (e.g. how seasonal effects were chosen for the Holt-Winters prediction model) and how goodness-of-fit was assessed.

**Response**: We thank the reviewer for this important question. The difference between a malaria case and a confirmed malaria case is based on the WHO document. Regarding the WHO malaria terminology, 2021 update, Geneva: World Health Organization; available at https://www.who.int/publications/i/item/9789240038400. The definitions are on page 8.

Regarding the reproducibility, as mentioned in lines 192–194, the Holt-Winters exponential smoothing method was used for time series forecasting. Since some months of our time series contain zero values (see data extract below), the **additive seasonal model** was selected over the multiplicative version, which cannot handle zeros due to its proportional nature.

|  | Jan | Feb | Mar | Apr | May | Jun | Jul | Aug | Sep | Oct | Nov | Dec |
| --- | --- | --- | --- | --- | --- | --- | --- | --- | --- | --- | --- | --- |
| 2010 | 2 | 0 | 0 | 3 | 0 | 1 | 2 | 1 | 1 | 5 | 6 | 8 |
| 2011 | 6 | 1 | 0 | 1 | 0 | 1 | 3 | 4 | 3 | 6 | 3 | 1 |
| 2012 | 1 | 1 | 5 | 0 | 0 | 2 | 2 | 0 | 2 | 9 | 6 | 7 |
| 2013 | 10 | 0 | 0 | 0 | 0 | 0 | 4 | 0 | 1 | 1 | 6 | 2 |
| 2014 | 2 | 1 | 1 | 1 | 4 | 1 | 5 | 2 | 2 | 1 | 0 | 0 |
| 2015 | 3 | 0 | 0 | 0 | 0 | 4 | 1 | 0 | 2 | 3 | 3 | 4 |
| 2016 | 1 | 2 | 1 | 4 | 4 | 4 | 3 | 3 | 3 | 0 | 1 | 1 |
| 2017 | 2 | 0 | 0 | 0 | 1 | 2 | 3 | 4 | 2 | 4 | 1 | 4 |
| 2018 | 0 | 1 | 2 | 1 | 0 | 2 | 1 | 0 | 3 | 5 | 1 | 2 |
| 2019 | 2 | 1 | 0 | 2 | 2 | 3 | 4 | 4 | 5 | 7 | 3 | 6 |
| 2020 | 5 | 1 | 1 | 0 | 0 | 0 | 0 | 0 | 0 | 0 | 3 | 0 |
| 2021 | 0 | 1 | 1 | 0 | 1 | 2 | 2 | 0 | 4 | 4 | 2 | 3 |
| 2022 | 0 | 0 | 0 | 1 | 0 | 1 | 0 | 4 | 5 | 4 | 4 | 8 |
| 2023 | 3 | 2 | 2 | 1 | 3 | 1 | 5 | 5 | 0 | 8 | 3 | 3 |
| 2024 | 1 | 1 | 1 | 0 | 1 | 3 | 2 | 5 | 2 | 4 | 1 | 5 |

To determine the seasonal structure, we adopted a 12-month periodicity — a standard assumption for monthly data — and confirmed its validity by observing a **recurring annual cycle** in the data. This pattern, with increasing imported cases especially during the second semester, is clearly visible in Figure 2 and in the decompose graphic (figure review 1) and reflects **seasonal waves linked to travel from endemic African regions**.

Figure review 1. Decomposition of Cape Verde’s imported malaria cases Additive time-series model.

Model adequacy was assessed through several diagnostics. The **Augmented Dickey-Fuller test** confirmed that the time series is stationary (p < 0.01); the **Shapiro-Wilk test** indicated the residuals are normally distributed (W = 0.989, p = 0.24) (figure review 2); Residual plots (figure review 3) showed no clear patterns, and the histogram resembled a normal distribution. These plots assess the temporal dependence between forecast components, which supports the evaluation of forecast stability and the coherence of prediction intervals over time.

Figure review 2. Residual Analysis of Cape Verde’s imported malaria cases Additive time-series model.

Figure review 3. Cross-correlation plots among forecasted point values and their associated 95% prediction intervals.

Therefore, the model provides a reasonably good fit and captures the seasonal trend well. We have included a full residual analysis in supplementary files, which supports the model’s suitability for forecasting in the public health planning and actions. We understand that this model is appropriate for advising the control actions by Cape Verde’s government.

Please submit your revised manuscript by Jun 08 2025 11:59PM. If you will need more time than this to complete your revisions, please reply to this message or contact the journal office at [globalpubhealth@plos.org](mailto:globalpubhealth@plos.org). When you're ready to submit your revision, log on to <https://www.editorialmanager.com/pgph/> and select the 'Submissions Needing Revision' folder to locate your manuscript file.

Please include the following items when submitting your revised manuscript:

- A rebuttal letter that responds to each point raised by the editor and reviewer(s). You should upload this letter as a separate file labeled 'Response to Reviewers'.
- A marked-up copy of your manuscript that highlights changes made to the original version. You should upload this as a separate file labeled 'Revised Manuscript with Track Changes'.
- An unmarked version of your revised paper without tracked changes. You should upload this as a separate file labeled 'Manuscript'.

Guidelines for resubmitting your figure files are available below the reviewer comments at the end of this letter.

Journal Requirements:

1. Please review your reference list to ensure that it is complete and correct. If you have cited papers that have been retracted, please include the rationale for doing so in the manuscript text, or remove these references and replace them with relevant current references. Any changes to the reference list should be mentioned in the rebuttal letter that accompanies your revised manuscript. If you need to cite a retracted article, indicate the article’s retracted status in the References list and also include a citation and full reference for the retraction notice.

**Response**: The authors confirm that the reference is complete and correct.

**Reviewer #1: Dear Authors,**

Thank you for the updates — they certainly enhance the clarity and overall quality of the manuscript.

I have one remaining comment:

I appreciate the inclusion of Table 1; however, I’m still unclear on the distinction between a Malaria case and a malaria case, confirmed. In both instances, it appears that any positive diagnostic test defines the case. Apologies if I’m overlooking something.

**Response**: Dear reviewer, Thank you for your comments. Yes, what defines a «malaria case» and a «malaria case confirmed» is a positive diagnostic test. The definition is based on the WHO document. In the case of the WHO malaria terminology, 2021 update, Geneva: World Health Organisation; available at https://www.who.int/publications/i/item/9789240038400, the definitions are on page 8, and the authors just transcribe the definition.

Reviewer #3: Thank you to the authors for their comprehensive responses and updates to the manuscript. All feedback has been appropriately incorporated, except the addition of study limitations in the discussion. On line 488, this section is introduced but does not cover any limitations.

**Response**: Dear reviewer, Thank you for your comments. We have updated the manuscript. Please see the new version, lines 494-503.

While the manuscript is generally well written, it requires another proofread as there remains some editing to be done (examples given below). Once this is complete, I recommend the paper for publication.


PROOF-READING EDITS

General: check for consistency in use of malaria vs Malaria throughout.
Line 112 - Suggest rephrasing to “Malaria surveillance data…” i.e. remove “It is also crucial to note…” as you make a similar assertion in line 116.
Line 120 - Typos: “decades” and “pre-elimination”

Line 142 - 142: Could you merge these? I.e. just say “...western region of the islands, which is more temperate and less mountainous.”
Line 180 - “It is…” not “It’s”
Line 186 - 187: Suggest rephrasing: “Table 1 provides definitions of key terms used in this study.”
Line 196: Authors use the term “National Malaria Control Program” and introduce the acronym at this point but have also referred to it as “National Malaria Program” earlier (e.g. line 173). Please check for consistency and introduce the acronym in the first instance.
Line 235: Is “anonymised” meant to be removed? If so, also remove the “and” before “publicly”.
Line 257 - 259: Suggest rephrasing for clarity “During the study period, there were 26 recrudescence cases. The majority (18/26) were reported during the 2017 outbreak, with sporadic recrudescence cases also reported in 2013, 2016, 2019 and 2020.”
Line 262: Do you mean “recrudescence” instead of “recurrences
Line 261 - 264: There seems to be a lot of duplication here. Please rewrite or remove duplicated sentences.
Table 2 - Please check for consistency of upper and lower case throughout the table (e.g. sometimes you use title case “Mortality Rate” and sometimes sentence case “Incidence rate”).
Line 282: Suggest removing this sentence or rephrasing to “The remaining months had between x% and 10.4% of the total annual reported cases.”
Line 295: Consider rephrasing to be more concise: “Throughout all years, the majority of cases were reported in Santiago Island (261; 68.1%). Praia, the capital, accounted for 50.4% (209/383) of imported cases nationwide and 80.0% (206/261) of those within Santiago Island (Fig 4).”
Line 326: Indigenous should not be capitalised.
Line 337: Perhaps rephrase to just list the most prominent countries of origin. Eg “ Guinea-Bissau, Angola, Senegal and Nigeria were the most common countries of origin for imported malaria cases.” You address the language connection lower down. Please check this interpretation is correct as there seem to be other countries with more imported cases.
Line 353 - 355: It seems like you’re saying these are risk factors and are also barriers to risk. Please rephrase.
Line 367: Remove “the facility of transport” and just start the sentence with “The increase or flights and mobility…”
Line 381: Remove “the” … i.e. “...between Cabo Verde and endemic malaria countries.”
Lines 382 and 386 - why is “importation of cases” in quotation marks?

**Response**: Dear reviewer, Thank you for your comments. All suggestions were adequately corrected. Please find attached a new version of the manuscript updated.

We hope these addresses and improvements or question. address the editor's and reviewer’s comments and questions. Anyway, the authors are still available for any additional clarification and/or question.
